# Temporally-coherent terawatt attosecond XFEL synchronized with a few cycle laser

**DOI:** 10.1038/srep37700

**Published:** 2016-11-28

**Authors:** Sandeep Kumar, Yong Woon Parc, Alexandra S. Landsman, Dong Eon Kim

**Affiliations:** 1 Department of Physics, Center for Attosecond Science and Technology, Pohang University of Science and Technology, Pohang, 37673, South Korea; 2 Max Planck Center for Attosecond Science, MPK, POSTECH, Pohang, 37673, South Korea; 3 Pohang Accelerator Laboratory, Pohang, 37673, South Korea; 4 Max Planck Institute for the Physics of Complex Systems, Noethnitzer Str. 38, 01187 Dresden, Germany

## Abstract

Attosecond metrology using laser-based high-order harmonics has been significantly advanced and applied to various studies of electron dynamics in atoms, molecules and solids. Laser-based high-order harmonics have a limitation of low power and photon energies. There is, however, a great demand for even higher power and photon energy. Here, we propose a scheme for a terawatt attosecond (TW-as) X-ray pulse in X-ray free-electron laser controlled by a few cycle IR pulse, where one dominant current spike in an electron bunch is used repeatedly to amplify a seeded radiation to a terawatt level. This scheme is relatively simple, compact, straightforward, and also produces a temporally and spectrally clean pulse. The viability of this scheme is demonstrated in simulations using Pohang accelerator laboratory (PAL)-XFEL beam parameters.

Over the past decade, the field of ultrafast laser physics has moved from its infancy to become a major force not only in atomic and chemical physics, but also at the interface of multiple areas of fundamental and applied science that involve imaging and control of electron dynamics^1^,^2^,^3^. Its progress was enabled by the creation of isolated attosecond pulses utilizing laser-based high-order harmonics (HOH)^4^,^5^,^6^,^7^. Since this attosecond pulse is naturally synchronized to a driving few-cycle IR pulse, the delay between the attosecond pulse and its driving laser pulse can be controlled with a high degree of precision, leading to a cornucopia of pump-probe experiments that capture electron dynamics in atoms, molecules and solids on the attosecond time-scale^8^,^9^,^10^,^11^.

Nonetheless, the low conversion efficiency of conventional laser-based HOH is a major limitation, which leads to low photon flux for the attosecond pulse and photon energy in extreme ultraviolet (XUV) region. X-ray free electron laser (XFEL) sources based on self-amplified spontaneous emission (SASE) scheme, on the other hand, offer unprecedented power, high peak brightness and coherence characteristics^12^,^13^,^14^. In addition, the higher photon energy (in the soft and hard X-ray range) matches the energy scales of core electrons inside atoms and molecules, giving access to previously unexplored phenomena. Hence, a great deal of theoretical effort has been aimed at shortening the duration of the XFEL pulse to sub-femtosecond levels^15^,^16^,^17^,^18^,^19^,^20^,^21^,^22^,^23^,^24^. Most of these theoretical proposals rely on only a small portion of electrons in phase space, thereby resulting in relatively low peak power. This limitation was overcome recently by Tanaka^25^, and Prat *et al*.^26^ who proposed a mechanism for a TW-attosecond X-ray pulse using a combination of slotted foil^15^, enhanced SASE (ESASE)^18^, and optical and/or electron beam delay between undulator sections. While these schemes offer power much beyond the conventional laser-based HOH sources, they lack synchronization with an external source which is essential for high resolution pump-probe experiments. Such a synchronization is necessary to scan the pump-probe delay with high precision, needed to capture ultra-fast electron dynamics.

This paper reports the first realization of an isolated TW attosecond X-ray pulse with an excellent temporal and spectral structure and synchronization to an external source, which is well suited to high precision ultrafast dynamics studies. The controlled interaction of a few cycle IR pulse with an electron bunch under a proper modulator and a chicane lead to a single, dominant electron spike^22^. This electron spike is fed into a series of short undulators, and tapering and a match between the radiation pulse and the electron spike is introduced for higher power and excellent temporal structure. This scheme is relatively simple in terms of implementation, as one has to match only one major current spike to one major radiation spike in undulator stages. In a few-cycle current modulation, where only one current spike is dominant, the alignment of the current spike with the seed radiation is easier compared to the case of many current-spikes.

Unlike other recent proposals, our set-up does not require a slotted foil which degrades the electron-beam quality. Our proposal uses a baseline configuration that could be implemented at PAL-XFEL^14^ or a similar existing XFEL facility. The current spike is well synchronized with the driving laser and hence produces an X-ray pulse which is synchronized with the driving few cycle pulse. Our method therefore combines the fine control over pump-probe delay with a high power (in the terawatt regime) offered by the XFEL sources.

## Method

### Basic layout: current modulation and amplification

**Figure 1:**
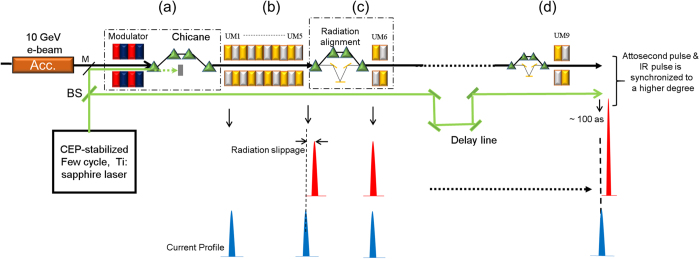
A schematic layout and working principle of attosecond-terawatt XFEL. A 10 GeV electron beam is modulated in energy and density by **(a)** modulator (wiggler) and chicane system. A CEP-stabilized few cycle, Ti: sapphire laser (1200 nm wavelength and 5 fs pulse-duration) is used for the electron beam energy modulation. **(b)** SASE undulator, consisting of 5 undulator modules, is used for seed radiation generation. **(c)** The chicane-mirror unit for radiation alignment followed by one undulator module for radiation amplification. **(d)** The units similar to **(c)** are repeated; only difference is that the chicane-mirror system is relatively smaller. In the bottom part, relative positions of current-spike (blue) and corresponding radiation-spike (red) are shown. If a portion of the modulation laser beam is picked up and used in a pump-pulse experiment with XEEL pulse, the synchronization between them is ensured.

Figure 1 shows the basic scheme along with its working mechanism. A 10 GeV (relativistic factor γ ~ 2 × 10^4^) electron-beam with a total electron bunch charge of 0.2 nC and an average current of 6 kilo-ampere (kA) is used. The electron bunch length is 35 fs long with a normalized emittance of 0.5 μm-rad and an energy spread of 0.5 MeV. Such an electron beam and an optical few-cycle laser of wavelength λ_*L*_ is injected synchronously inside a modulator, as shown in Fig. 1a. The interaction of the electron beam with the laser pulse in the modulator produces an energy modulation, imprinting the shape of optical cycles of the laser field on the energy spectrum of the electron beam. The modulator with two wiggler periods is used. The FEL resonance condition, 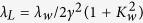 is satisfied, where λ_*L*_ is the laser wavelength, λ_*w*_ the wiggler period (50 cm in this study), γ the electron beam relativistic factor. *K*_*w*_ is the wiggler parameter given by *eB*_0_λ_*w*_/2π*mc*, where, *e* and *m* are the charge and mass of the electron, *c* velocity of light and *B*_0_ the magnet field. The electron beam enters a dispersive chicane, which induces strong bunching at *λ*_*L*_. As a result, the current profile contains a few current spikes, the number of which can be varied depending on a laser wavelength, a laser pulse duration and the energy spectrum of an electron beam^22^. The simulations were carried out for different laser wavelengths, pulse durations, pulse energies, and chicane dispersion factor *R*_56_ to maximize the peak current modulation and the contrast ratio of the main peak magnitude to the side peak magnitudes. To generate this electron beam distribution, the six-dimensional particle tracking code ELEGANT^27^ was used.

This density-modulated electron beam is then sent to the first undulator line, consisting of 5 undulator modules (UMs) (Fig. 1b) for SASE radiation. Due to high-current at the main electron spike, stronger radiation amplification is achieved in a shorter length of an undulator. For undulator radiation, simulations are performed using three-dimensional time-dependent FEL code GENESIS^28^. The saturation length inversely depends on the electron beam current. Therefore, the XFEL output due to the radiation of the major current spike will saturate earlier than in the normal SASE operation without the current spike. We choose the first undulator section to be shorter than the saturation length for the major spike, in order to avoid the saturation of the radiation from the main current spike and to minimize the degradation of the electron beam quality.

### Radiation alignment

In an undulator, the radiation travels ahead of electrons by one radiation wavelength λ_r_ per undulator period (λ_*u*_) and slips ahead by *Nλ*_*r*_, where *N* is the total number of undulator periods passed. To compensate this radiation slippage, a chicane-mirror system, consisting of four-dipole magnets and a set of plane mirrors (Fig. 1c), is introduced between undulator modules^29^. The chicane delays the electron bunch and dilutes the microbunching developed in undulator modules during SASE radiation while the reflective mirror system gives a temporal delay to the radiation relative to the electron bunch. For the next stage of amplification, a short UM is used to minimize the radiation slippage and pulse broadening. This UM can be added several times to get a clean single amplified pulse (Fig. 1d). Our simulations show that the amplification can be made even to a TW level within a reasonable numbers of UMs (9 UMs in this study).

## Results and Discussion

To demonstrate the performance of the proposed scheme, we present simulation results, based on PAL-XFEL parameters^14^, for the case of attosecond-TW XFEL at 12.4 keV or 0.1 nm in the hard X-ray regime.

**Figure 2:**
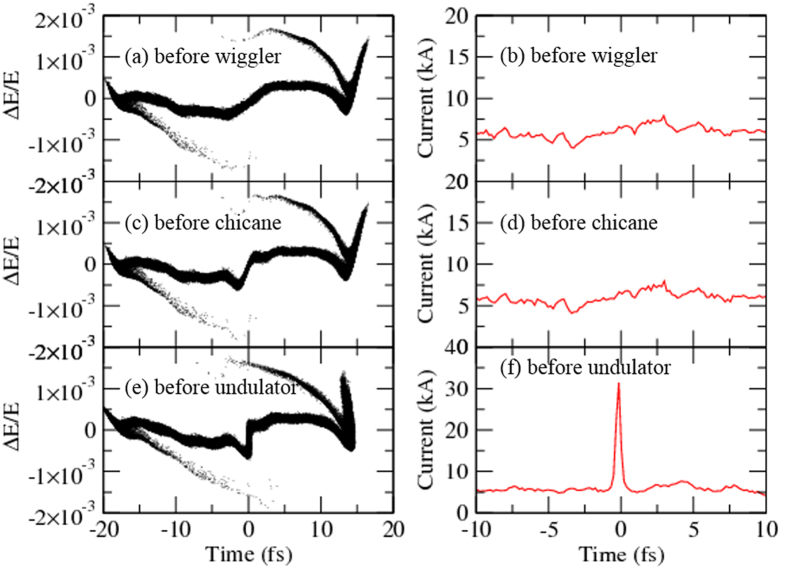
Longitudinal energy distribution and current profile of a 10 GeV electron beam at three different locations. (**a,b**) Before wiggler, **(c,d)** before chicane, **(e,f)** before undulator (Fig. 1a for the locations). A single current spike of 33 kA with a base current of 6 kA is generated for radiation in UMs.

Figure 2 is the energy and density modulation of the electron bunch by a 1200 nm, 5 fs FWHM and 0.13 mJ carrier-envelop-phase (CEP) stabilized laser focusing at the center of the modulator and by a chicane, respectively. Figure 2a,c,e show the electron-beam longitudinal phase-space distribution and Fig. 2b,d,e show the current distribution at the entrance of the wiggler, chicane and undulator, respectively. The central part of the current distribution has only one single current spike with a peak current of 33 kA (Fig. 2f), almost 6 times larger than the background. This large difference in current between the central peak and the background leads to a significant difference in the radiation output power at the end between them, as discussed below. To obtain this current modulation, the momentum compaction factor R_56_ of magnet chicane (Fig. 1a) is optimized to be 0.16 mm with a bending angle of the dipole 0.32^0^, a drift length of 2.5 m between the first and second dipole magnet and also a length of 2.5 m between the third and fourth dipole magnet. Each dipole magnet is 0.3 m long. This electron bunch is then sent to a series of UMs. Note that the current enhanced SASE scheme not only enhances the peak current but also increases the energy spread at the current peak position, which has been taken into account in this FEL simulations.

**Figure 3:**
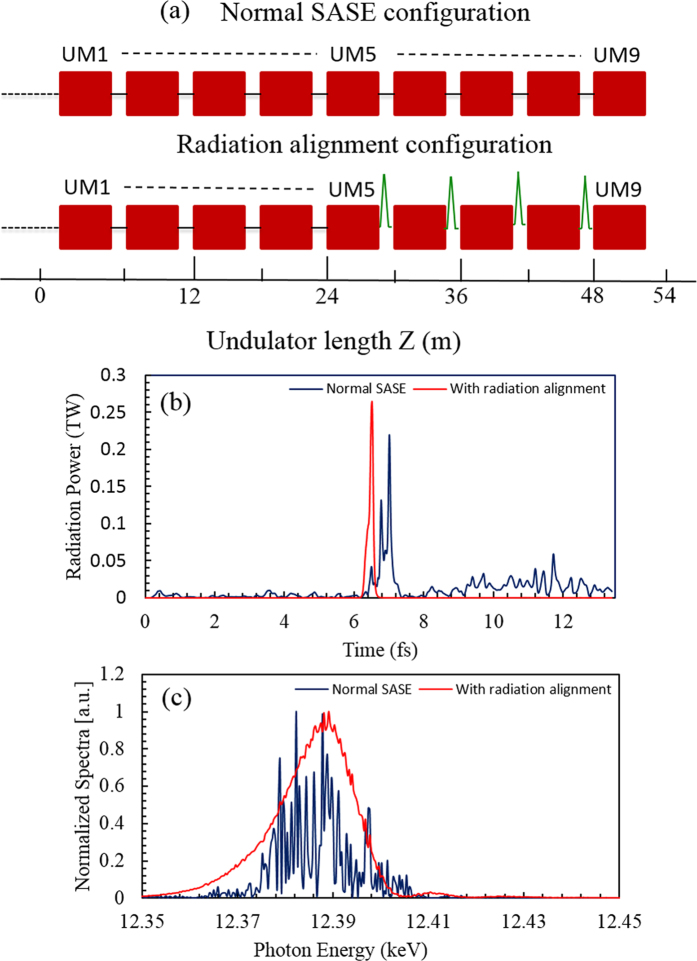
Two undulator configurations and amplification. (**a**) Two undulator configurations; the upper one is used for normal SASE and the lower-one in case of radiation alignment. For radiation alignment, one chicane-mirror unit is inserted between undulator modules. Total 9 UMs are considered for radiation generation in both configurations. **(b)** The temporal profiles of the radiation pulse after 9 UMs; blue-line for the normal SASE case, red-line for the radiation alignment case. **(c)** Corresponding radiation spectra; the spectrum in case of radiation alignment (red-line) is clean while that for normal SASE case (blue-line) is noisy.

Figure 3a shows two undulator configurations used in our scheme. The first one is the normal SASE configuration and the second is the radiation alignment configuration in which chicane-mirror setups are inserted between UMs in the later part of the undulators. Each UM is 6-m long (5-m for the undulator itself and 1-m for a drift section). The undulator period is 26 mm. In total, 9 UMs are used in the undulator line. Figure 3b shows the temporal profiles of the radiation for the case of normal SASE (blue line) and radiation alignment (red-line) taken after the 9^th^ UM (~50 m in total) along with the corresponding spectra (Fig. 3c). Since the radiation always slips ahead of the electrons by one radiation wavelength, this net slippage of the radiation results in several spikes, leading to broadening of the radiation pulse, as shown by blue-line in Fig. 3b.

To control this radiation slippage, radiation alignment setups are introduced between UMs. It is done only for a few UMs in the latter part of the undulators for the sake of simplicity. The idea is to use the main current spike repeatedly for a few UMs to keep the excellent temporal profile, control the pulse broadening, and enhance the radiation power. The maximum power obtained in the normal SASE configuration is 0.2 TW and the main spike width is almost 300 as FWHM with several other radiation spikes. This spiky temporal profile of SASE (blue line) is due to the growth of synchrotron side bands generated due to the continuous interaction of the radiation with the fresh part of the electron bunch. To assess the importance of radiation alignment, the simulation is repeated. The red-line shows the temporal profile when the radiation alignment is used. After 9 UM amplification, the radiation alignment gives a clean attosecond pulse of 100 as FWHM with 0.3 TW power. One can see a big improvement in the temporal structure. The increment in radiation power is marginal. Figure 3c shows the corresponding power spectrum. The spectrum also gets cleaner in case of the radiation alignment compared to the normal SASE spectrum. In the radiation alignment configuration, the radiation pulse is aligned with the electron beam after the first 5 UMs. A magnet chicane similar to the self-seeding chicane at PAL-XFEL^30^ is chosen. The magnet chicane, consisting of 0.1 m long dipole magnets with a magnetic field strength of 1.0T, is able to cover the electron beam delay needed in the amplifier section. The drift space between two neighboring UMs is enough to accommodate the radiation-delay chicane used for radiation alignment. Note that the radiation-delay after each UM is adjusted to align the seed radiation with the main current spike to maximize the peak power and the contrast ratio of the main radiation spike with the background in the next UM. The required radiation-delay after each undulator module is equivalent to the sum of the net radiation slippage in the undulator module and the electron-beam delay. Because of large energy modulation, the high peak current (33 kA) suffers from energy-chirp due to space-charge effects. There are two kinds of space charge effects of electron beam: short-range and long-range space charge effects. The short-range space-charge working against microbunching has been taken care of by the GENESIS^28^ code used in our simulation. However, the longitudinal debunching effect is presently not included in GENESIS^28^ code. The longitudinal debunching causes a reduction in the peak current; however, it is less severe at higher electron beam energies (i.e. 10 GeV) where the reduction in peak current is of the order of a few percent only^31^. Additionally, the tapering used in our simulation should help to minimize the space charge effects^19^,^32^.

**Figure 4:**
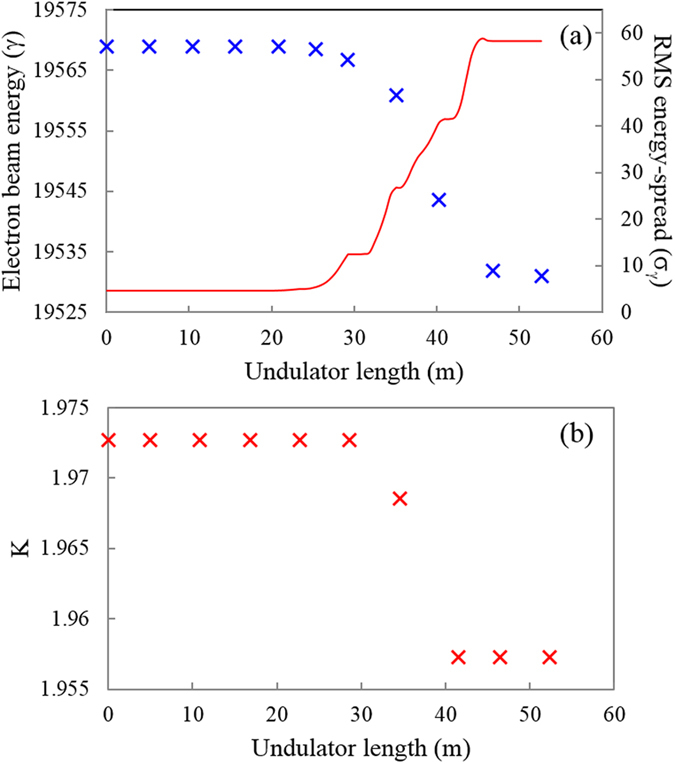
Change of electron beam energy (γ) and its RMS energy spread (σ_γ_) at the major current spike and tapering for compensation. (**a**) γ and σ_γ_ variation along the undulator length. (**b**) The tapering of magnetic field along 9 UMs.

Another concern in this scheme is the degradation of beam quality, such as the energy spread, because of repeated use of the major current spike. Figure 4a shows the energy loss (γ) at the major current spike (in blue ‘×’) along the undulator length. Also, corresponding RMS energy-spread, σ_γ_ (the root means square value of the energy distribution in terms of electron rest mass energy) is shown on the secondary y-axis (red line). At the entrance of UM1, σ_γ_ is 4.5 and increases up to 12 after 5 UMs amplification (~30 m). This degradation of beam quality weakens the amplification in the downstream undulator section. Therefore, to compensate the degradation and to increase the power growth efficiency, a tapering^33^ to the UMs after the first 5 UMs is introduced, preserving the resonance condition. Figure 4b reveals the tapering applied along the UMs. The first 5 UMs have the same undulator parameter K. The tapering is applied to only the last 4 UMs. The values for undulator parameter K of the last 4 UMs is optimized to sustain the FEL resonance condition and to maximize the radiation power.

**Figure 5:**
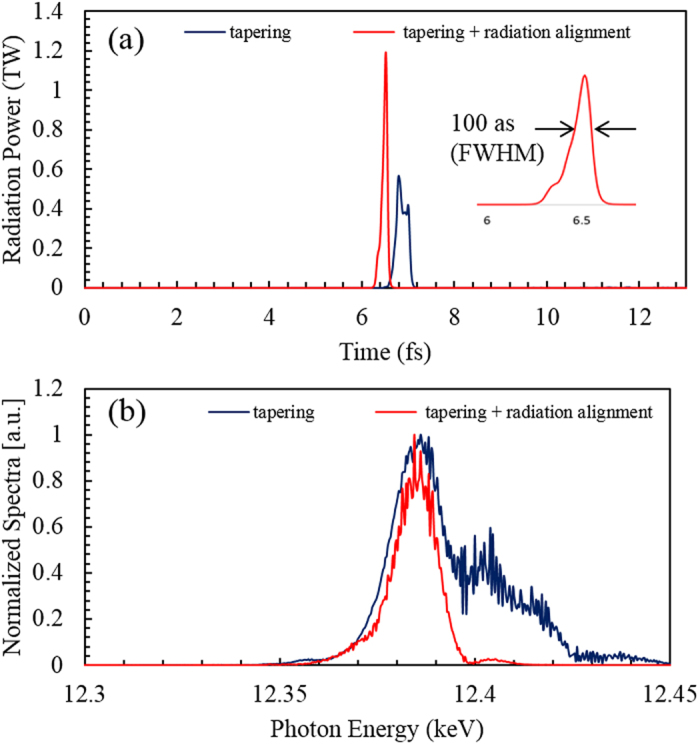
Temporal and spectral profile of attosecond pulse after 9 UM amplification. (**a**) For tapering case (blue-line), radiation pulse duration is 300 as with 0.55 TW power. For the tapering with radiation alignment (red-line), the pulse duration is 100 as with 1.2 TW power, the inset shows the enlarged view of 100 as pulse. **(b)** Corresponding radiation spectra; the tapering with radiation alignment (red-line) gives a cleaner spectrum compared to the tapering case (blue-line).

A further simulation for similar parameters with tapering has been carried out. Figure 5 shows the simulation results for radiation power and spectrum at the end of UMs for two cases; (a) tapering only, where the main radiation spike power is increased up to 0.56 TW and pulse-width is 300 as FWHM. (b) By adding the radiation alignment to the tapering, the radiation power is enhanced up to 1.2 TW and the pulse-width reduced to 100 as FWHM. Comparing the tapering only case (blue-line) in Fig. 5a with normal SASE (blue-line) of Fig. 3a, one can see that with the tapering, the growth of side bands can be suppressed to a significant degree so that the temporal and spectral profile are improved as shown in Fig. 5a,b. They are still spiky. An even more important feature of this scheme is the very clean temporal and spectral profile (red line). To check whether the final pulse is Fourier limited or not, we calculate the frequency-time bandwidth relation Δ*v*Δ*τ* = *c*Δ*λ*Δ*τ/λ*^2^ which is ~0.441 for Gaussian pulse. For tapering and optical alignment case, the pulse-width is Δ*τ* ~ 110 as FWHM. From the spectrum profile, for ΔE ~20 eV at λ = 0.1 nm, the product Δ*v*Δ*τ* is 0.499 slightly larger than 0.441 that shows that the final pulse is nearly Fourier-transform limited.

**Figure 6:**
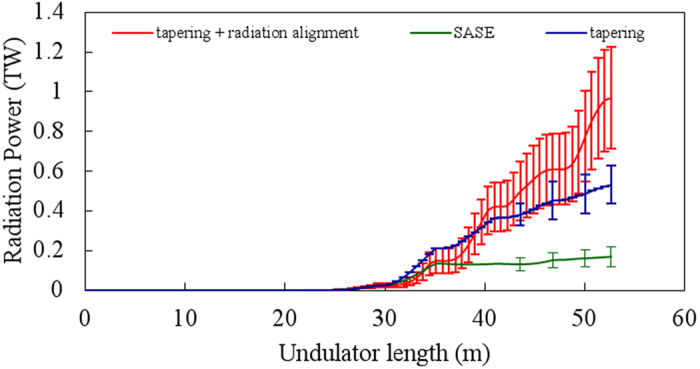
The average radiation power and power fluctuations for 10 different random seeds are shown for three cases. The red line relates to the case of the tapering with radiation alignment showing an output power of 1.01 ± 0.36 TW. The blue line relates to the case of tapering case only with an output power of 0.5 ± 0.11 TW. The green-line corresponds to normal SASE case with an output power of 0.17 ± 0.1 TW.

In a further simulation, we have run 10 different simulations using different seeds due to the shot noise of the electron beam. Figure 6 shows the radiation power versus the undulator length with these statistical fluctuations. It is clearly demonstrated that the radiation output improves from the normal SASE case to the tapering case to tapering with radiation alignment, as more controls added to UMs. The simulation result for the case of the tapering with radiation alignment shows that the average power after 9 UM stage amplification (red-curve) is 1.0 ± 0.4 TW (90 ± 36 μJ), while the average pulse duration 94 ± 28 as FWHM. It is worthwhile to mention that in the case of tapering with and without radiation alignment, the power is not saturated yet up to 50 meter, implying that if more UMs are added, the radiation power is expected to increase further. Therefore, using our scheme; we could get ≥1 TW, 100 as FWHM with an excellent temporal and spectral profile by the combination of tapering and radiation alignment. In a conventional SASE FEL (no current spikes), for given similar parameters, only 30 GW power at 12.4 keV photon energy is expected at saturation. After UMs, using radiation alignment and tapering together, the main radiation-pulse finally evolves into an isolated intense X-ray pulse with a peak power of ≥1 TW and a pulse duration of 100 as FWHM at the same photon energy. All these considerations indicate that our scheme is superior in getting a clean radiation pulse with TW level power in attosecond time scale.

### Synchronization and stability

Synchronization between an X-ray pulse and an optical laser pulse is of vital importance to perform well-defined pump-probe experiments. Timing stability between FEL X-ray pulses and independent external optical laser pulses is important. In ESASE operation, the arrival of an electron-bunch and an optical laser pulse at the entrance of a modulator should match properly. Here, since a 40 fs-long electron bunch and 5 fs-long optical lasers are considered, the relative timing jitter between the electron beam and laser should be controlled below ~35 fs. If a longer electron beam is used, this requirement can be relaxed accordingly. An achievable timing jitter between the electron beam and the modulation laser is shown at the 20-fs level^34^. In an ESASE operation, an X-ray pulse is generated by the electrons that interact with the laser pulse, and hence are naturally synchronized to it.

However, for the realization of this scheme, only an inherent synchronization is not sufficient. We also need to take care of synchronization at each chicane-mirror setup where the electron beam energy jitter and the timing jitter between the electron beam and the modulation laser could affect the performance of our scheme. The electron beam energy jitter mainly affects the timing after passing through chicanes. After first chicane-mirror setup, the timing jitter will be converted to current-spike position jitter. Hence, a stable electron beam with an energy jitter below 0.02% is desirable for this scheme.

In our scheme, after a 30 m long undulator, the first magnet-chicane (R_56_ ~ 20 μm) changes the electron position by *s*_1_−*s*_0_ = *R*_56_ Δ*E/E* ~ 10*nm* for Δ*E/E* ~ 0.05%. In the time domain, this corresponds to Δ*t* = (*R*_56_/*c*) Δ*E/E* ~ 33 as, which is much smaller than the current spike width 400 as. After the first magnet-chicane, the radiation pulse-width for seeding is around 0.4 fs FWHM. Therefore, the shift of 33 as in current position can be compensated with the fine adjustment of magnet-chicane. This adjustment of magnet chicane is quite small compared to the electron beam delay, and should not affect the electron beam current distribution. For the remaining three magnet-chicanes which are shorter (R_56_ = 4 μm), the change of electron position s_1_−s_0_ is 5 nm, 8 nm, and 11 nm, respectively. In the time domain, Δt is 16.5 as, 26.5 as, and 36 as, which is quite small compared to current spike width 400 as FWHM. In total, four magnet-chicanes are used. The net displacement in current spike position is ~34 nm (current spike width 120 nm FWHM). Therefore, even though the original shape of the current spike is smeared out after 4 magnet-chicanes, this smearing is not significant in our scheme. The change of 10 nm in electron’s position is enough to destroy microbunching. However, the microbunching develops rapidly in the next undulator section due to high current peak of e-bunch and strong seed pulse power. Hence the good amplification is still maintained in each stage.

**Figure 7:**
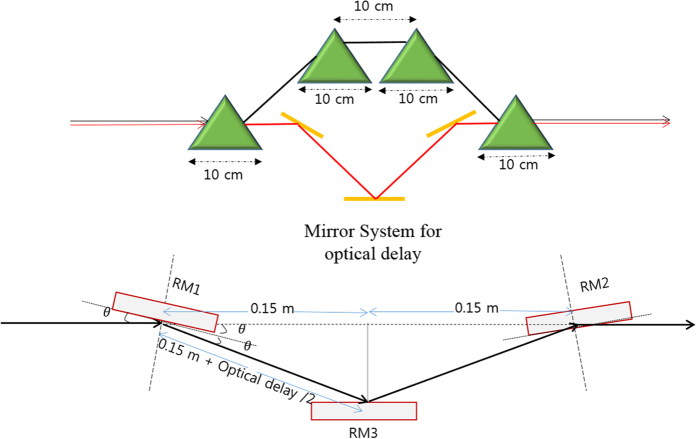
Schematic layout for X-ray delay system.

The schematic layout of X-ray delay system is shown in Fig. 7. The X-ray delay system includes a magnetic-chicane and a set of reflected mirrors^25^. For its practical design, the optical components have to meet various requirements such as a high reflectivity, delay with sub-fs resolution, a large delay range, and the wide photon energy range of XFEL (1–12 keV). These properties have to be achieved with a minimal disturbance of the beam position and direction, a high mechanical stability, making a temporal resolution of 100 as or so feasible. For x-ray delay system, instead of a monochromator as in the self-seeding scheme, a set of reflective mirrors is installed to give a temporal delay to the radiation to align with the main current peak of the electron bunch.

The first magnet-chicane is used after a 30 m long SASE undulator whose R_56_ is almost 20 μm that gives an electron beam delay of around 10 μm. While a relatively shorter magnetic chicane is used after a short undulator (~ 5m long undulator), the chicane accomplishes three tasks. It creates an offset for the installation of mirrors, it removes microbunching developed in the previous undulator and it acts as delay line for electron beam. For a given geometry, the total length is ~0.7 m, an e-beam is delayed by 2 μm. The net radiation slippage is 0.019 μm (0.063 femtoseconds), and total optical delay is 2.019 μm. To create such an optical-delay, the mirror system as shown in Fig. 7 is needed. The length of the mirror is estimated by the expression *D*sin*θ* = *d*, where D is the mirror’s half-length and d is the radiation beam radius. For a mirror deflection of *θ* = 0.1^0^ and a radiation beam size of *d* = 50 *μm*, D turns out to be 2.73 centimeter. Therefore, a mirror with a length of 5~6 cm (~2D) and a deflection angle of 0.1^0^ would be enough in the shorter chicane-mirror system to obtain the required optical delay mentioned earlier. Mirror stages with 10 nm resolution are commercially available. Hence, the delay can be controlled with a high degree of precision

In pump-probe experiments, the mechanical vibration leads to jittering in the optical path lengths. It is demonstrated that at FEL experimental stations, the timing jitter generated due to a free space propagation of ~100 m and due to folding mirrors can be controlled with 5 fs accuracy via currently available technology^34^,^35^.

By using a feedback loop^36^ for all the mirrors, i.e. both optical and X-ray mirror, such mechanical vibration can be suppressed and the time delay between pump (optical laser) and probe pulse (X-ray) can be controlled within 20 attosecond RMS. Thus, if the feedback loop technology^36^ is added to the mirror-chicane system, then an XFEL pulse might even be synchronized with current spike and with optical laser on the attosecond time-scale. Combining all of these technologies together, a synchronization in 100 attosecond range between a pump laser and an X-ray pulse is achievable with the rapid developing technology in near future.

The result of this study indicates a feasible method to generate a TW attosecond pulse in XFEL, which is synchronized with a few-cycle IR pulse and can be readily used in pump-probe experiments that capture ultra-fast electron dynamics in atoms, molecules, nano materials and bulk solids. To demonstrate the applicability of our method in the hard X-ray regime, we produce an isolated 100 attosecond FWHM, 1 TW pulse at 12.4 keV (~0.1 nm) within a 50 m long undulator. This attosecond X-ray pulse has excellent temporal and spectral structure properties. Our scheme is straight-forward to implement and can be adapted to the existing FEL facilities and also leads to a substantial reduction in cost due to the use of a short undulator.

## Additional Information

**How to cite this article**: Kumar, S. *et al*. Temporally-coherent terawatt attosecond XFEL synchronized with a few cycle laser. *Sci. Rep.* **6**, 37700; doi: 10.1038/srep37700 (2016).

**Publisher's note:** Springer Nature remains neutral with regard to jurisdictional claims in published maps and institutional affiliations.
